# Construction and validation of a prediction model for early recurrence after catheter ablation in patients with persistent atrial fibrillation based on BNP, Ang Ⅱ, homocysteine, MHR, NLR

**DOI:** 10.3389/fcvm.2025.1589351

**Published:** 2025-05-16

**Authors:** Yuning Chen, Guojian Sun, Zhijun Zhu, Farong Shen

**Affiliations:** ^1^ Department of Cardiovascular Medicine, Zhejiang Qiushi Cardiovascular Hospital, Hangzhou, China; ^2^ Department of Cardiovascular Medicine, Inner Mongolia People’s Hospital, Hohhot, China

**Keywords:** persistent atrial fibrillation, catheter ablation, early recurrence, prediction models, serum indicators

## Abstract

**Objective:**

To explore the feasibility and clinical value of early recurrence prediction model for persistent atrial fibrillation after catheter ablation based on brain natriuretic peptide (BNP), angiotensin Ⅱ(Ang Ⅱ), homocysteine, monocyte-to-high density lipoprotein cholesterol (MHR) and neutrophil-to-lymphocyte ratio (NLR).

**Methods:**

279 patients with persistent atrial fibrillation who underwent catheter ablation in our hospital from January 2022 to December 2024 were divided into training group (*n* = 195) and verification group (*n* = 84) according to the ratio of 7:3. The risk factors were screened by univariate and multivariate Logistic regression analysis, and the nomogram model was constructed, and the effectiveness of the model was evaluated in the verification set.

**Results:**

Multivariate Logistic regression analysis showed that diabetes history, elevated BNP, homocysteine, MHR, NLR and Ang Ⅱ were independent risk factors for early postoperative recurrence (*P* < 0.05). The C-index index of the nomogram model in the training set and the verification set are 0.803 and 0.846, respectively, and the AUC under the ROC curve is 0.802 (95% CI: 0.685–0.918) and 0.855 (95% CI: 0.736–0.973), respectively.

**Conclusion:**

The nomogram prediction model can well predict the early recurrence risk of patients with persistent atrial fibrillation after catheter ablation, and provide a reference for clinical individualized treatment, but it needs further verification by large sample and multi-center research.

## 1 Introduction

Atrial fibrillation (AF) is a common arrhythmia that significantly increases the risk of heart failure and thromboembolism, severely impacting patients' quality of life (1). Catheter ablation is a key treatment for persistent AF, but early recurrence limits its effectiveness (2). Therefore, accurately predicting early recurrence after catheter ablation is crucial. In recent years, studies have shown that various biomarkers and inflammatory indicators, like BNP, Ang Ⅱ, and homocysteine, are closely linked to the occurrence, development, and prognosis of atrial fibrillation. BNP reflects cardiac function, Ang Ⅱ is crucial for atrial fibrillation's electrical and structural remodeling, and elevated homocysteine increases cardiovascular disease risk. In addition, inflammatory indexes such as the ratio of monocytes to high-density lipoprotein cholesterol (MHR) and neutrophils to lymphocytes (NLR) are also related to the occurrence and development of atrial fibrillation (3, 4). Although there have been many studies on atrial fibrillation—related risk factors, research on constructing an early recurrence prediction model for patients with persistent atrial fibrillation after catheter ablation using BNP, Ang Ⅱ, homocysteine, MHR, and NLR indicators is relatively scarce (5). There are few studies on the prediction model of early recurrence of patients with persistent atrial fibrillation after catheter ablation combined with BNP, Ang Ⅱ and other indicators. The reason is that in the past, more attention was paid to a single or a few indicators, and when multiple indicators were comprehensively analyzed, differences in detection methods and interpretation of clinical significance, which made it difficult to integrate them, and the complexity of patients' conditions, making it difficult to screen the combination of key synergistic indicators. The purpose of this study is to fill this gap. By analyzing the relationship between indicators and recurrence, a prediction model is constructed and verified, which provides an effective tool for clinic and improves the prognosis and overall treatment level of patients.

## 2 Materials and methods

### 2.1 Subjects

A total of 279 patients with persistent atrial fibrillation who received catheter ablation in our hospital from January 2022 to December 2024 were selected as the research subjects. Inclusion criteria: ① Patients met the diagnostic criteria of persistent atrial fibrillation (6), i.e., the duration of atrial fibrillation exceeded 7 days; ② Aged 18–80 years old; ③ Sign informed consent form and voluntarily participate in this study. Exclusion criteria: ① patients with severe hepatic and renal insufficiency (Child-Pugh C grade of liver function or EGFR of renal function < 30 ml/min/1.73 m); ② Combined with malignant tumor (including but not limited to solid tumors and hematological tumors, and receiving radiotherapy and chemotherapy or targeted therapy during the study period); ③ Has a recent (within 3 months) history of acute myocardial infarction and stroke; ④ There is an active stage of infectious diseases (such as active tuberculosis, suppurative infection, etc.); ⑤ Active stage with autoimmune disease (such as active systemic lupus erythematosus, rheumatoid arthritis, etc.); ⑥ Those who failed to cooperate in completing the follow-up. Patients were divided into a training set (*n* = 195) and a verification set (*n* = 84) in a 7:3 ratio using a completely randomized approach. Sample size calculation: According to previous studies (7), the early recurrence rate after catheter ablation in patients with persistent atrial fibrillation is approximately 20%–30%. The expected recurrence rate for this study is 25%, with a set test power of 80% (*β* = 0.2) and a significance level of 0.05 (*α* = 0.05). Using G * Power software, a minimum sample size of 260 is required for univariate logistic regression analysis. This study included a total of 279 patients, with a sufficient sample size. Proportional risk hypothesis test: In multivariate logistic regression analysis, the Schoenfeld residual test is used to evaluate the proportional risk hypothesis. The results showed that the Schoenfeld residuals of each variable were not significantly correlated with time (*P* > 0.05), indicating that the proportional hazards hypothesis was valid and suitable for logistic regression analysis.

### 2.2 Data collection

General clinical data such as age, gender, height, weight, body mass index (BMI), hypertension, diabetes, coronary heart disease, smoking, and alcohol consumption were collected. Fasting venous blood was collected preoperatively and tested for BNP, Ang Ⅱ, homocysteine, MHR (monocyte count/high-density lipoprotein cholesterol), NLR (neutrophil count/lymphocyte count), C-reactive protein (CRP), D-dimer, fibrinogen, left atrial diameter (LAD), left ventricular ejection fraction (LVEF), glycated hemoglobin (HbA1c), total cholesterol (TC), triglycerides (TG), high-density lipoprotein cholesterol (HDL-C) and low-density lipoprotein cholesterol (LDL-C). Record operation-related information, including operation method, operation time, and ablation strategy. The detection of interleukin-6 (IL-6) is not included in the analysis because it is not a routine preoperative examination item in our center, but it can be considered as a supplement to inflammatory markers in future research.

### 2.3 Postoperative follow-up

Follow-up within 1 and 3 months postoperatively to determine whether atrial fibrillation recurs through outpatient, telephone, or dynamic electrocardiogram examination. Recurrence of atrial fibrillation within 3 months after surgery is defined as early recurrence.

### 2.4 Statistical methods

SPSS 26.0 and R language 4.5.3 were used for statistical analysis. The measurement data were expressed by mean standard deviation (normal distribution) or median [*M*(Q1, Q3)] (non-normal distribution), and the comparison between groups was conducted by independent sample t test or Mann–Whitney *U* test. Counting data were expressed by the number of cases (%), and the comparison between groups was made by *χ* test or Fisher exact test. Independent predictors were screened by single factor and multivariate Logistic regression (stepwise method, *α* in =0.05, *α* out =0.10), and odds ratio (OR) and 95% confidence interval (CI) were calculated. The discrimination of the model was evaluated by receiver operating characteristic curve area (AUC), and the calibration was tested by Hosmer-Lemeshow and analyzed by calibration curve. Decision curve analysis (DCA) was used to evaluate the clinical practicability. *P* < 0.05 is statistically significant.

## 3 Results

### 3.1 Comparison of general clinical features of patients between training set and verification set

There was no significant difference in general clinical characteristics such as age, gender, BMI and most laboratory indicators between the two groups (*P* > 0.05), as shown in Table 1.

**Table 1:** Comparison of general clinical characteristics of patients between training set and verification set.

Index		Training set (*n* = 195)	Validation set (*n* = 84)	Statistical values	*P*
Age (years)		55.38 ± 8.72	56.28 ± 8.45	0.798	0.425
BMI (kg/m^2^)		22.46 ± 3.12	22.78 ± 3.05	0.791	0.429
Gender	Man	118 (60.51)	52 (61.90)	0.047	0.826
	Woman	77 (39.49)	32 (38.10)		
Smoking history	Have	85 (43.59)	36 (42.86)	0.012	0.909
	Without	110 (56.41)	48 (57.14)		
Drinking history	Have	72 (36.92)	33 (39.29)	0.139	0.708
	Without	123 (63.08)	51 (60.71)		
History of diabetes	Have	64 (32.82)	27 (32.14)	0.012	0.911
	Without	131 (67.18)	57 (67.86)		
History of hypertension	Have	102 (52.13)	44 (52.38)	0.001	0.992
	Without	93 (47.69)	40 (47.62)		
History of coronary heart disease	Have	58 (29.74)	27 (32.14)	0.159	0.689
	Without	137 (70.26)	57 (67.86)		
BNP (pg/ml)		256.34 ± 78.45	248.67 ± 75.89	0.756	0.450
Ang Ⅱ (pg/ml)		45.67 ± 12.34	44.89 ± 11.98	0.488	0.625
Homocysteine (μmol/L)		14.56 ± 3.45	14.23 ± 3.12	0.753	0.451
MHR		0.45 ± 0.12	0.44 ± 0.11	0.654	0.513
NLR		2.34 ± 0.78	2.29 ± 0.75	0.496	0.619
CRP (mg/L)		5.67 ± 2.34	5.45 ± 2.12	0.740	0.459
D-dimer (mg/L)		0.78 ± 0.23	0.76 ± 0.21	0.683	0.494
Fibrinogen (g/L)		3.45 ± 0.89	3.42 ± 0.87	0.260	0.795
LAD (mm)		42.34 ± 5.67	41.89 ± 5.46	0.614	0.539
LVEF (%)		58.34 ± 6.78	57.89 ± 6.41	0.516	0.605
HbA1c (%)		6.45 ± 1.23	6.39 ± 1.18	0.378	0.705
TC (mmol/L)		4.51 ± 1.15	4.52 ± 1.16	0.066	0.947
TG (mmol/L)		1.78 ± 0.55	1.74 ± 0.54	0.560	0.575
HDL-C (mmol/L)		1.23 ± 0.34	1.21 ± 0.32	0.458	0.646
LDL-C (mmol/L)		2.89 ± 0.78	2.85 ± 0.75	0.397	0.691

### 3.2 Univariate analysis of risk factors for early recurrence after catheter ablation with a training set

There were 32 cases (22.22%) of early postoperative recurrence in the training group and 13 cases (20.97%) of early postoperative recurrence in the verification group. Single factor analysis showed that there were significant differences in indicators such as diabetes history, BNP, homocysteine, MHR, NLR, Ang Ⅱ, and CRP between patients with and without early recurrence (*P* < 0.05). In the regression model, the tolerance of each variable was > 0.1, VIF was < 10, and condition index was < 30. In addition, the proportion of variances of multiple covariates without the same feature value was more than 50%. Hence, there was no collinearity of each covariate, as shown in Table 2.

**Table 2:** Univariate analysis of risk factors for early recurrence after catheter ablation with training set.

Index		Early recurrence (*n* = 32)	No early recurrence (*n* = 163)	Statistical values	*P*
Age (years)		57.85 ± 8.45	56.87 ± 8.51	0.596	0.551
BMI (kg/m^2^)		23.14 ± 3.01	22.78 ± 3.11	0.607	0.548
Gender	Man	20 (62.50)	98 (60.12)	0.063	0.801
	Woman	12 (37.50)	65 (39.88)		
Smoking history	Have	15 (46.88)	70 (42.94)	0.168	0.681
	Without	17 (53.12)	93 (57.06)		
Drinking history	Have	12 (37.50)	60 (36.81)	0.005	0.940
	Without	20 (62.50)	103 (63.19)		
History of diabetes	Have	18 (56.25)	46 (28.22)	4.817	0.028
	Without	14 (43.75)	85 (52.15)		
History of hypertension	Have	21 (65.63)	81 (49.69)	2.721	0.099
	Without	11 (34.37)	82 (50.31)		
History of coronary heart disease	Have	10 (31.25)	48 (29.45)	0.041	0.838
	Without	22 (68.75)	115 (70.55)		
BNP (pg/ml)		284.67 ± 85.34	245.79 ± 75.23	2.613	0.009
Ang Ⅱ (pg/ml)		48.55 ± 13.45	42.52 ± 12.12	2.526	0.012
Homocysteine (μmol/L)		16.78 ± 3.67	15.27 ± 3.25	2.351	0.019
MHR		0.52 ± 0.15	0.41 ± 0.13	4.264	0.001
NLR		2.67 ± 0.84	2.24 ± 0.75	2.906	0.004
CRP (mg/L)		6.28 ± 2.45	5.41 ± 2.16	2.036	0.043
D-dimer (mg/L)		0.87 ± 0.60	0.74 ± 0.30	1.841	0.067
Fibrinogen (g/L)		3.67 ± 0.95	3.41 ± 0.84	1.586	0.119
LAD (mm)		44.87 ± 5.89	43.22 ± 5.32	1.575	0.116
LVEF (%)		56.77 ± 7.87	58.31 ± 7.28	1.079	0.281
HbA1c (%)		6.87 ± 1.35	6.52 ± 1.25	1.429	0.154
TC (mmol/L)		4.87 ± 1.26	4.45 ± 1.16	1.846	0.064
TG (mmol/L)		1.98 ± 0.65	1.85 ± 0.58	1.136	0.257
HDL-C (mmol/L)		1.13 ± 0.35	1.24 ± 0.34	1.665	0.097
LDL-C (mmol/L)		3.12 ± 0.84	2.85 ± 0.78	1.767	0.078

### 3.3 Multivariate logistic regression analysis

The presence of early recurrence was used as the dependent variable (0 = no early recurrence, 1 = presence of early recurrence) and the factor *P* < 0.05 in the univariate analysis was used as the covariate for further multivariate Logistic regression analysis (variable assignment table is shown in Table 3). The results showed that diabetes history, BNP, homocysteine, MHR, NLR, and Ang Ⅱ were the independent risk factors for early recurrence in patients with persistent atrial fibrillation after catheter ablation (*P* < 0.05). See Table 4.

**Table 3:** Variable assignment method.

Variable	Meaning	Evaluation
X1	History of diabetes	0 = none, 1 = yes
X2	BNP	Continuous variable
X3	Homocysteine	Continuous variable
X4	MHR	Continuous variable
X5	NLR	Continuous variable
X6	Ang Ⅱ	Continuous variable
X7	CRP	Continuous variable
Y	Early relapse	Early recurrence = 1, no early recurrence = 0

**Table 4:** Multivariate analysis of poor clinical effect of training set.

Factor	*B*	Standard error	Wald	*P*	OR	95% Confidence interval
History of diabetes	1.239	0.477	6.750	0.009	3.452	1.356–8.788
BNP	0.009	0.003	7.845	0.005	1.009	1.003–1.016
Homocysteine	0.182	0.076	5.774	0.016	1.199	1.034–1.391
MHR	4.797	1.714	7.837	0.005	121.153	4.214–3,483.017
NLR	1.023	0.332	9.491	0.002	2.783	1.451–5.336
Ang Ⅱ	0.038	0.019	3.996	0.046	1.038	1.001–1.077
Constant	−15.205	2.764	30.262	0.001	0.001	

### 3.4 Construction of nomogram prediction model

**Figure 1:**
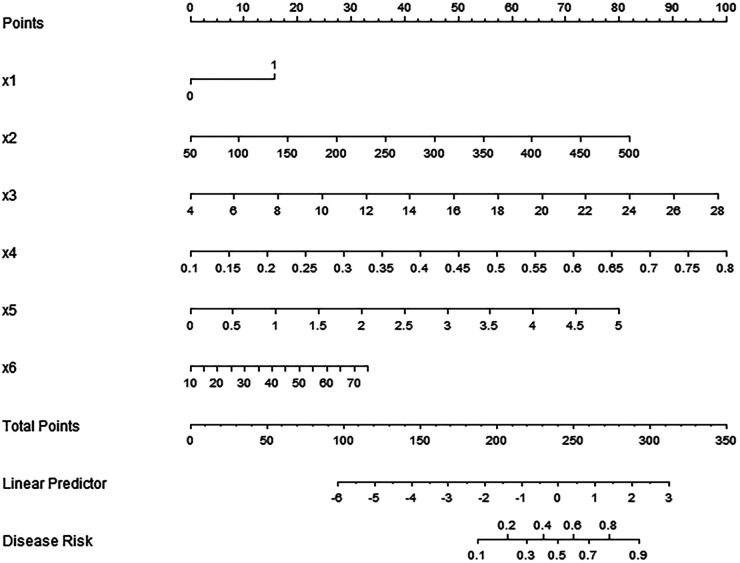
Nomogram prediction model for early recurrence after catheter ablation in patients with persistent atrial fibrillation. x1, history of diabetes; x2, BNP; x3, homocysteine; x4, MHR; x5, NLR; x6, Ang Ⅱ.

Based on the independent risk factors determined by multivariate Logistic regression analysis, a nomogram prediction model for early recurrence after catheter ablation in patients with persistent atrial fibrillation was constructed. Each independent risk factor in the model was scored, and the total score for predicting early postoperative recurrence was calculated, expressed as the predicted recurrence probability, as shown in Figure 1.

### 3.5 Assessment and validation of nomogram prediction model for early recurrence after catheter ablation

**Figure 2:**
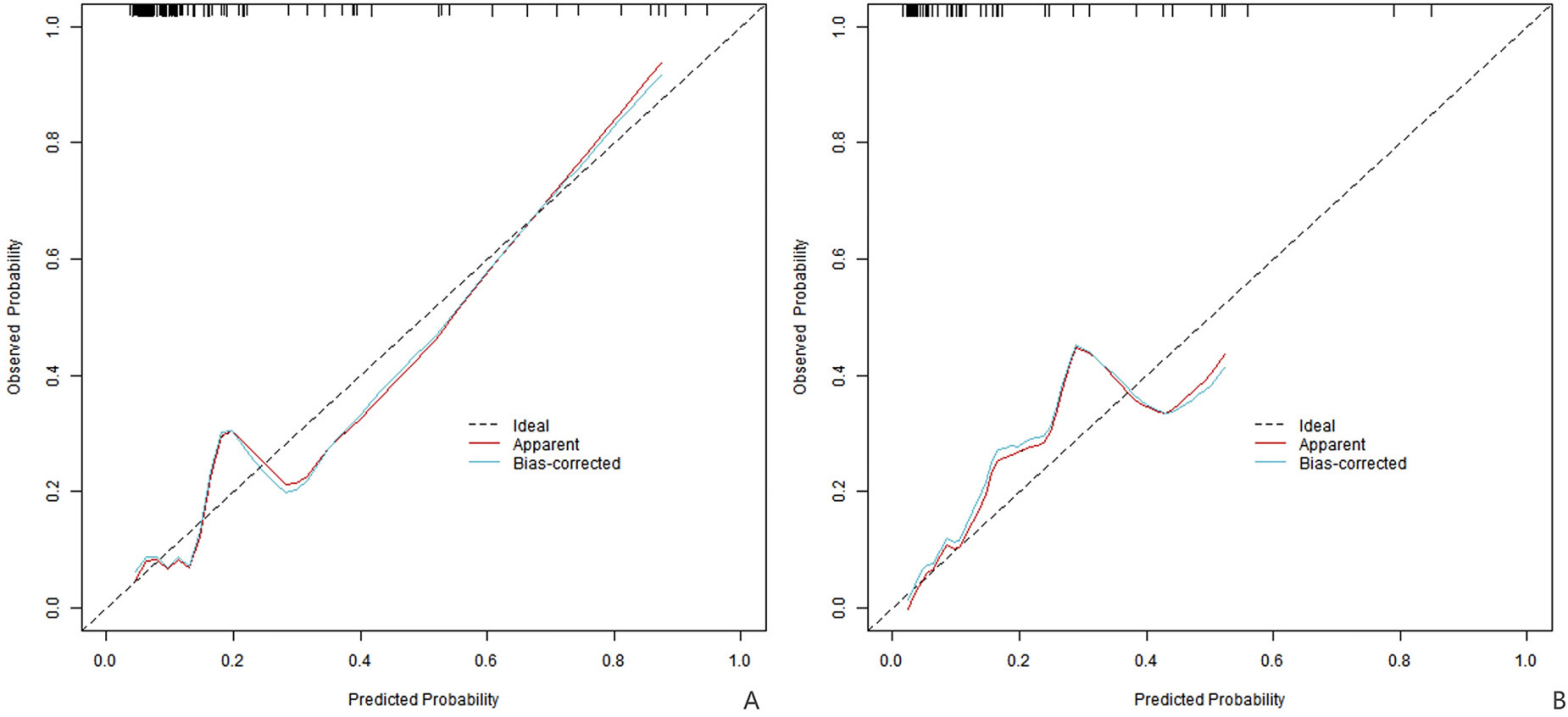
Calibration curve (**A** is the training set, and **B** is the verification set).

**Figure 3:**
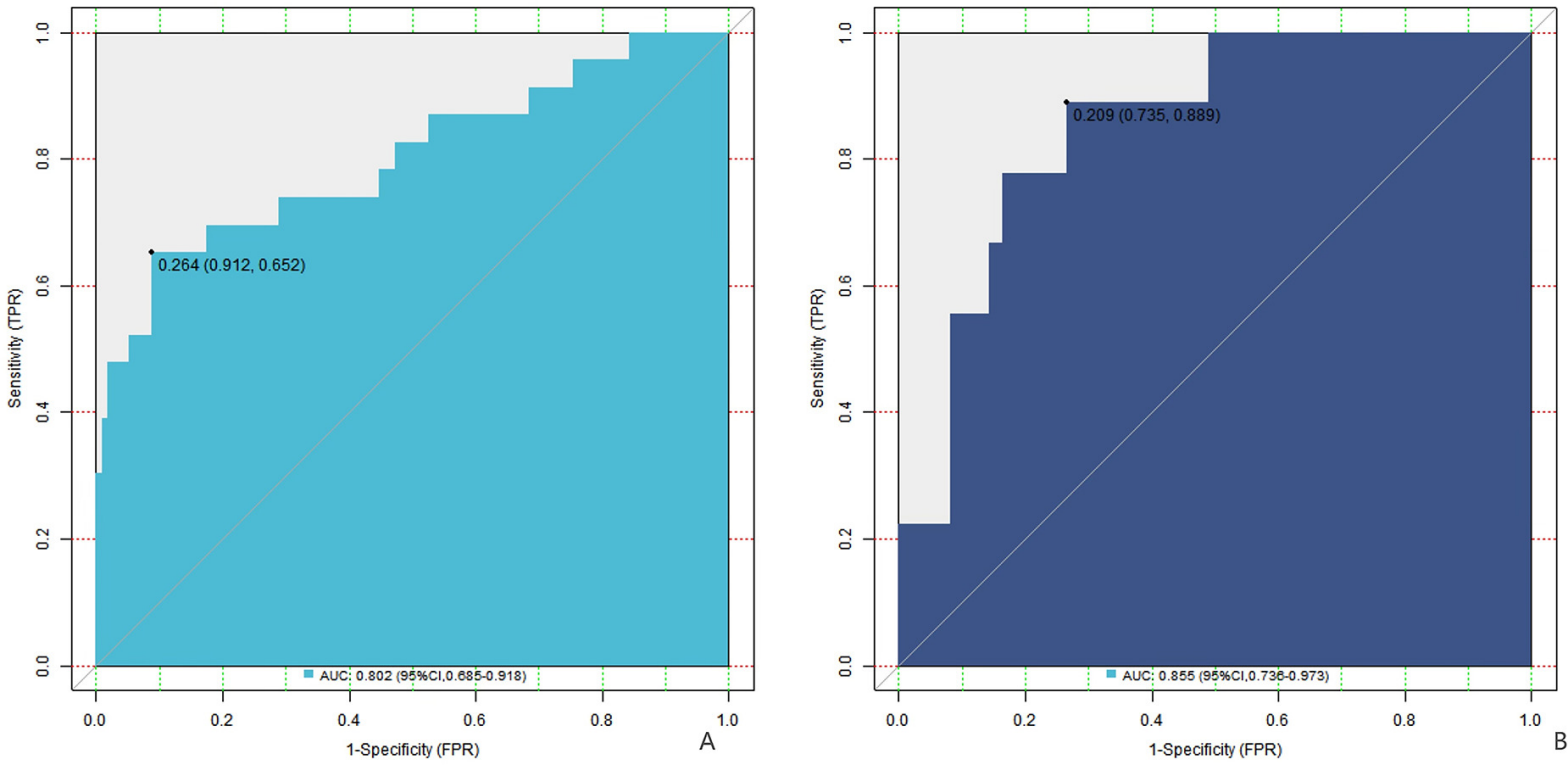
ROC curve (**A** is the training set, and **B** is the verification set).

C-index index (consistency index) is an important index to measure the distinguishing ability of forecasting models, and its value ranges from 0.5 (no forecasting ability) to 1 (perfect forecasting). In the training set, the C-index index of the nomogram prediction model was 0.803, the average absolute error of the predicted value in accordance with the actual value shown in the calibration curve was 0.095, and the *P* = 0.244 test by Hosmer-Lemeshow indicated that the model fitted well. The ROC curve showed that the model had an AUC of 0.802 (95% CI: 0.685–0.918) for predicting early postoperative recurrence, a sensitivity of 0.652, and a specificity of 0.912. In the validation set, the C-index was 0.846, the mean absolute error was 0.099, the Hosmer-Lemeshow test *P* = 0.523, the AUC was 0.855 (95% CI: 0.736–0.973), the sensitivity was 0.778, and the specificity was 0.816. The C index of this model in the training set and the validation set is 0.803 and 0.846 respectively, which indicates that when doctors use this model to predict recurrence, there is a 80.3%–84.6% probability that they can correctly distinguish between recurrent and non-recurrent patients (better than 50% of random guess). The area under ROC curve further proves that its discrimination ability is close to excellent level (AUC > 0.8). The calibration curve and ROC curve are shown in Figures 2, 3, respectively.

### 3.6 Analysis of decision curve of early recurrence nomogram prediction model after catheter ablation

**Figure 4:**
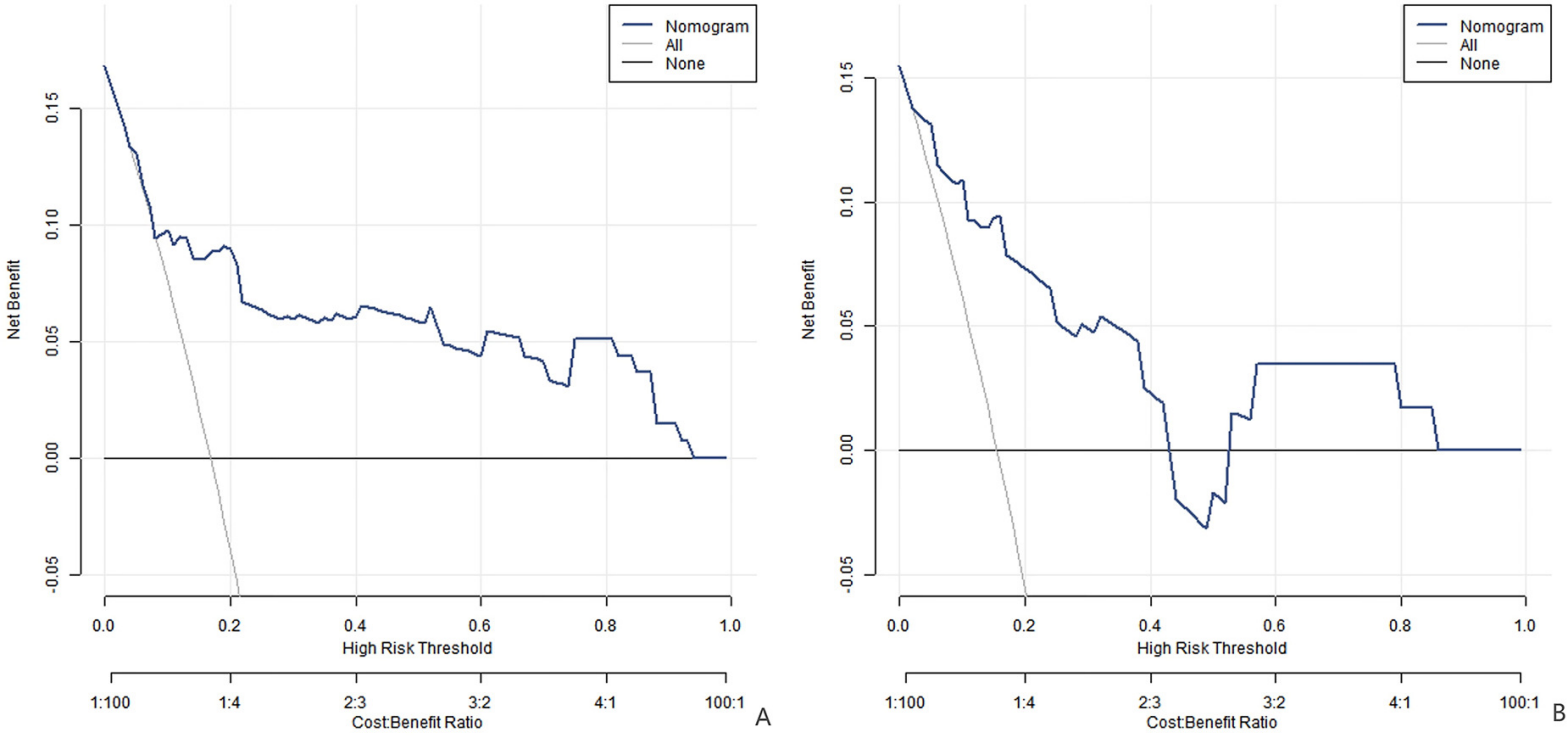
Decision curve (**A** is the training set, and **B** is the verification set).

Analysis of decision curve showed that when the threshold probability was between 0.08 and 0.85, the application of the nomogram model constructed in this study to predict the early recurrence of persistent atrial fibrillation in patients after catheter ablation was more clinically beneficial than the preoperative decision that all patients would relapse or all patients would not relapse. In simpler terms, the decision curve helps doctors understand that within this range of probability, using our model to predict early recurrence can offer more useful information for making treatment decisions. For example, it can help doctors better identify patients at high risk of recurrence, so as to take more targeted preventive measures. This means that the model can provide valuable reference for clinicians and help them make more accurate treatment plans, so as to improve the treatment effect and the prognosis of patients, as shown in Figure 4.

## 4 Discussion

Atrial fibrillation (AF) is a very common clinical arrhythmia. Its persistent attack significantly heightens the risk of serious complications like heart failure and thromboembolism, severely impairing patients' quality of life and having an adverse impact on prognosis. Catheter ablation is currently the key treatment for persistent atrial fibrillation. Although it brings hope for rehabilitation for many patients, the early postoperative recurrence seriously restricts the therapeutic effect. The purpose of this study was to construct and verify a prediction model for early recurrence of persistent atrial fibrillation after catheter ablation based on brain natriuretic peptide (BNP), angiotensin II (Ang II), homocysteine, monocyte-to-high density lipoprotein cholesterol (MHR) and neutrophil-to-lymphocyte ratio (NLR), so as to provide reference for clinical prevention and treatment.

The results of this study showed that the early postoperative recurrence rates in the training set and the verification set were 22.22% and 20.97%, respectively, which were similar to the early recurrence rates after catheter ablation of atrial fibrillation reported in previous studies (7). Diabetes, a common metabolic disease, can cause myocardial cell metabolic disorder, myocardial fibrosis, and cardiac autonomic neuropathy due to long—term hyperglycemia. These changes affect the heart's structure and function, increasing the risk of atrial fibrillation recurrence (8). BNP, secreted by ventricular myocytes, reflects cardiac function impairment when elevated. During atrial fibrillation, heart structure and function changes stimulate increased BNP secretion. Elevated BNP not only reflects the heart's current state but also affects the RAAS system, increasing cardiac load, altering myocardial electrophysiological characteristics, and promoting atrial fibrillation recurrence (9). An increase in homocysteine is closely related to cardiovascular disease risk. It can damage vascular endothelial cells through oxidative stress and inflammatory reactions, affecting cardiac function and increasing the likelihood of atrial fibrillation recurrence (10). The specific mechanisms include the production of reactive oxygen species, the reduction of NO production, and the triggering of vasodilation dysfunction; activating inflammatory cytokines, such as TNF-α and IL-6, affects the electrophysiological activity and structure of the heart (11). MHR and NLR are new inflammation indicators. The increase of MHR reflects the imbalance between inflammation and anti-inflammatory mechanism, and the increase of NLR indicates the activation of inflammation. Both of them are independent risk factors for early recurrence after catheter ablation, suggesting that inflammatory reaction plays an important role in postoperative recurrence of atrial fibrillation (12, 13). When MHR rises, monocytes release inflammatory mediators, changing the myocardial microenvironment, affecting electrophysiological stability, and promoting cardiac remodeling. When NLR rises, neutrophils release MPO and other substances, causing oxidative stress and damaging myocardial tissue. Lymphocytes, involved in immune regulation, have their number and function changes affecting the inflammatory reaction process, jointly increasing the risk of atrial fibrillation recurrence (14). Ablation—induced tissue injury triggers neutrophil infiltration, releasing ROS and MMP-9, destroying myocardial cell connections, and promoting fibrosis. After monocytes differentiate into macrophages, they secrete IL-6 and TNF-α, which up-regulate collagen synthesis and increase atrial matrix stiffness. This inflammatory microenvironment will also change the expression of ion channels, prolong the duration of action potential and form the basis of reentry. In addition, the decrease of HDL-C in MHR will weaken its anti-inflammatory function, further amplify the inflammatory damage, and lead to the increase of electrical conduction heterogeneity in the ablation scar area, which will become an important matrix for recurrence. Ang Ⅱ has multiple biological effects in the cardiovascular system. It can promote myocardial cell hypertrophy and fibrosis, leading to cardiac structural remodeling. Meanwhile, it can affect the electrophysiological properties of the heart, and promote the occurrence and maintenance of atrial fibrillation (15). Its mechanism involves activating MAPK pathway, inducing gene expression changes in myocardial cells, increasing the volume of myocardial cells, increasing myocardial cell volume and extracellular matrix, and affecting ion channel function, like enhancing L-type calcium current, which changes the electrophysiological characteristics of myocardial cells and promotes the occurrence and maintenance of atrial fibrillation. In this study, the increase of Ang Ⅱ is closely related to postoperative recurrence, and inhibiting its activity may reduce the risk of recurrence and provide a new target for clinical treatment (16, 17).

In this study, a nomogram prediction model was established to visually present the relationship between risk factors and early postoperative recurrence. The nomogram integrates multiple risk factors, assigns scores to each factor, and calculates the total score to predict the probability of disease or event (18). The model has good calibration and prediction efficiency in the training set and verification set, with C-index of 0.803 and 0.846 respectively, but there is still room for improvement in prediction ability, potentially due to single center, small sample and failure to include multiple factors. Future research should conduct large—sample, multi—center prospective studies, incorporate more potential influencing factors, and strengthen external verification. The area under ROC curve (AUC) of nomograph model is 0.802 (95% CI: 0.685–0.918) and 0.855 (95% CI: 0.736–0.973), with specificities and sensitivities of 0.652, 0.912, 0.778 and 0.816, respectively. The decision—curve analysis shows that when the threshold probability is between 0.08 and 0.85, the model has more clinical benefits than the traditional decision-making, and can assist clinicians to formulate reasonable treatment plans according to the patient's situation, improve the treatment effect and improve the prognosis. In clinical application, doctors can detect BNP, Ang Ⅱ and other indicators before operation, and combine the history of diabetes to calculate the recurrence risk by nomogram model. Specifically for the preoperative workflow, once the recurrence risk is calculated using the nomogram, it can serve as a basis for tailoring the surgical approach. For example, in high—risk patients, surgeons might choose a more comprehensive ablation strategy or allocate more time for the procedure to potentially reduce the recurrence risk. Also, anesthesiologists can adjust the anesthesia plan according to the risk level, ensuring a more stable peri—operative state. Regarding patient counseling, the nomogram provides a visual and quantitative tool. Doctors can show patients the specific score and predicted recurrence probability based on their individual data. This helps patients better understand the potential risks associated with their condition. For instance, a patient with elevated BNP and a history of diabetes can clearly see how these factors contribute to a higher recurrence risk through the nomogram. This understanding can empower patients to actively participate in their treatment. They may be more motivated to adhere to the recommended health management plans, such as strict control of blood sugar levels for diabetic patients. Moreover, it can also help patients make more informed decisions about additional treatment options, like whether to start anti—arrhythmic medications earlier or intensify anticoagulation therapy. For high-risk patients, we can increase the dosage of antiarrhythmic drugs, change or combine drugs, strengthen anticoagulation therapy, formulate strict health management plans, such as regular work and rest, moderate exercise, smoking cessation and alcohol restriction, and closely monitor the condition, check ECG every week within 1 month after operation, respond to recurrence in time, optimize nursing, and improve the quality of life and long-term prognosis of patients.

However, this study has limitations. First, the single-center retrospective design may limit the generalizability of findings, as patient demographics and clinical practices could differ in other settings. External validation in multicenter cohorts is needed to confirm our model's robustness. Second, the follow-up period (median 12 months) might be insufficient to capture late recurrences, which are clinically relevant in persistent AF. Third, despite rigorous statistical adjustments, potential selection bias could persist due to the exclusion of patients with incomplete data (*n* = 15). Future studies with prospective designs, longer follow-up, and broader inclusion criteria would strengthen these findings. Additionally, other factors that may affect the postoperative recurrence of atrial fibrillation, like gene polymorphism and lifestyle, were not considered in this study (19). Finally, the lack of external validation in this study, which may affect the reliability and applicability of the model. External validation is crucial for evaluating prediction models. Validating the model in different research populations can more accurately assess its generalization ability and reliability. However, due to time and resource constraints, external validation was not performed for this study. In future studies. Future studies should further expand the sample size, conduct multi-center prospective studies, incorporate more influencing factors, and conduct external verification to improve the prediction efficiency and reliability of the model (20). In addition, some studies pointed out that the blank period was significantly shortened after pulsed field ablation (PFA), which may have an impact on the results of this study (21). This technological shift may impact our model's predictive accuracy, as PFA alters both the definition of early recurrence and inflammatory response dynamics. While our model incorporates inflammatory markers (MHR, NLR) that are sensitive to tissue injury, PFA's reduced trauma and faster inflammatory resolution may modify their predictive trajectories. Notably, PFA shortens the blanking period to 1 vs. 3 months for conventional ablation, necessitating potential adjustments to both the early recurrence timeframe and risk factor weights. Key implications include: (1) Patients previously classified as early recurrences (1–3 months) might be reclassified, potentially changing risk factor profiles; (2) Inflammatory markers may demonstrate different predictive relationships due to PFA's accelerated healing; and (3) The model's current 3-month framework could overestimate recurrence risk in PFA cases. Future validation should therefore: (a) Test the model in PFA-treated cohorts, (b) Recalibrate early recurrence criteria to reflect the 1-month blanking period, and (c) Reassess the weighting of inflammatory indicators in this new context. It is worth noting that the confidence interval for MHR is extremely wide, suggesting potential instability in the estimate. This may be related to the relatively small sample size and the distribution of data within our study population. A larger sample size and more diverse data distribution could help stabilize the estimate and narrow the confidence interval in future studies.

In conclusion, the nomogram prediction model based on BNP, Ang Ⅱ, homocysteine, MHR and NLR has certain predictive value for the early recurrence risk of patients with persistent atrial fibrillation after catheter ablation, and providing a reference for making personalized treatment plans in clinic. However, in view of the fact that the prediction performance reflected by the C index value of this research model is not excellent, it is necessary to carry out large-sample and multi-center research, incorporate more influencing factors, conduct long-term follow-up and external verification, further improve the prediction efficiency and reliability of the model, and better serve the clinical prevention and treatment of persistent atrial fibrillation.

## Data Availability

The raw data supporting the conclusions of this article will be made available by the authors, without undue reservation.
